# Genetic diversity and signatures of selection of drug resistance in *Plasmodium* populations from both human and mosquito hosts in continental Equatorial Guinea

**DOI:** 10.1186/1475-2875-12-114

**Published:** 2013-03-27

**Authors:** Cristina Mendes, Patrícia Salgueiro, Vicenta Gonzalez, Pedro Berzosa, Agustin Benito, Virgílio E do Rosário, Bruno de Sousa, Jorge Cano, Ana Paula Arez

**Affiliations:** 1 Centro de Malária e outras Doenças Tropicais, Unidade de Parasitologia Médica, Instituto de Higiene e Medicina Tropical, Universidade Nova de Lisboa, Lisboa, Portugal; 2 Centro Nacional de Medicina Tropical, Instituto de Salud Carlos III, Madrid, Spain; 3 Centro de Malária e outras Doenças Tropicais, Unidade de Saúde Internacional, Instituto de Higiene e Medicina Tropical, Universidade Nova de Lisboa, Lisboa, Portugal

**Keywords:** Malaria, Equatorial Guinea, Genetic diversity, Drug resistance, *pfcrt*, *pfdhps*, *pfdhfr*, *pfmdr1*, Microsatellites, *Plasmodium falciparum*

## Abstract

**Background:**

In *Plasmodium*, the high level of genetic diversity and the interactions established by co-infecting parasite populations within the same host may be a source of selection on pathogen virulence and drug resistance. As different patterns have already been described in humans and mosquitoes, parasite diversity and population structure should be studied in both hosts to properly assess their effects on infection and transmission dynamics. This study aimed to characterize the circulating populations of *Plasmodium* spp and *Plasmodium falciparum* from a combined set of human blood and mosquito samples gathered in mainland Equatorial Guinea. Further, the origin and evolution of anti-malarial resistance in this area, where malaria remains a major public health problem were traced.

**Methods:**

*Plasmodium* species infecting humans and mosquitoes were identified by nested-PCR of chelex-extracted DNA from dried blood spot samples and mosquitoes. Analysis of *Pfmsp2* gene, anti-malarial-resistance associated genes, *Pfdhps*, *Pfdhfr*, *Pfcrt* and *Pfmdr1*, neutral microsatellites (STR) *loci* and *Pfdhfr* and *Pfdhps* flanking STR was undertaken to evaluate *P. falciparum* diversity.

**Results:**

Prevalence of infection remains high in mainland Equatorial Guinea. No differences in parasite formula or significant genetic differentiation were seen in the parasite populations in both human and mosquito samples. Point mutations in all genes associated with anti-malarial resistance were highly prevalent. A high prevalence was observed for the Pfdhfr triple mutant in particular, associated with pyrimethamine resistance.

Analysis of *Pfdhps* and *Pfdhfr* flanking STR revealed a decrease in the genetic diversity. This finding along with multiple independent introductions of *Pfdhps* mutant haplotypes suggest a soft selective sweep and an increased differentiation at *Pfdhfr* flanking microsatellites hints a model of positive directional selection for this gene.

**Conclusions:**

Chloroquine is no longer recommended for malaria treatment in Equatorial Guinea but sulphadoxine-pyrimethamine (SP) remains in use in combination with artesunate and is the only drug recommended in preventive chemotherapy in pregnancy. The high prevalence of point mutations in *Pfdhfr* and *Pfdhps* points to the danger of an eventual reduction in the efficacy of SP combined therapy in *P. falciparum* populations in Equatorial Guinea and to the essential continuous monitoring of these two genes.

## Background

Malaria continues to be one of the main public health problems in the world, affecting 106 countries, with approximately 216 million cases resulting in 650,000 yearly deaths [1]. This parasitic disease involves three living entities with complex interactions among them and transmission of *Plasmodium* parasites by their anopheline vectors is a crucial factor determining the epidemiology of malaria in endemic areas.

The level of genetic diversity of natural populations of *Plasmodium* is well demonstrated and both inter- and intra-specific mixed infections in the same host are common, especially in highly endemic areas [2]. The ecological interactions that these different and co-infecting parasite populations establish among them may be a source of selection on pathogen traits such as virulence and drug resistance.

Parasite genetic diversity and population structure in both humans and mosquitoes should be assessed in order to better determine the influence of different parasite populations on infection and transmission dynamics. In fact, both different associations of *Plasmodium* species as well as marked differences in the multiplicity of infection and allele diversity of *Plasmodium falciparum* populations were previously reported [3]. Furthermore, a recent analysis of both human peripheral blood samples and mosquitoes from the same location has revealed a completely unexpected picture related to the presence of *Plasmodium vivax* in an area where it had not yet been reported [4]. Differences have also been found in drug-resistant associated genes. In Gabon, Mharakurwa *et al*[5] reported that parasites in humans presented high levels of pyrimethamine (PYR)-resistant mutants, whereas parasites in *Anopheles* mosquitoes showed high levels of cycloguanil-resistant mutants.

For a period of time, the genetic diversity of *P. falciparum* populations has mainly been investigated through the analysis of mutation in polymorphic surface antigen coding genes [6,7]. However, this approach poses some limitations as it is impossible to know whether observed patterns reflect population history or natural selection [8]. Microsatellite sequences (STR), spread throughout the genome, are currently the neutral markers most commonly used to differentiate *P. falciparum* populations as these markers (short repeated nucleotide sequences) often present high levels of inter- and intra-specific polymorphism, particularly when the number of repetition is 10 or higher.

In Equatorial Guinea, malaria remains the major endemic disease and the leading cause of child mortality and morbidity. In recent years, the prevalence of infection has been reduced significantly on the Insular Region due to an effective vector control [1,9] whilst the prevalence of infection remains above 50% in children under five years old in mainland region [10]. Along with the high prevalence of infection, the dissemination of *P. falciparum* drug resistance still remains the main constraint to control malaria transmission in most endemic areas. Anti-malarial resistance has largely been studied through the analysis of mutations on several target genes associated with resistance to specific drugs, e g, *Pfcrt*[11] and *Pfmdr1*[12] linked to chloroquine (CQ) resistance; and *Pfdhfr*[13] and *Pfdhps* genes [14] associated with resistance to pyrimethamine (PYR) and sulphadoxine (SFX), respectively.

Increasing failure rates (40-50%) for CQ and around 25% resistance to sulphadoxine/pyrimethamine (SP) in under-five children was reported in 2003 in Malabo, the capital city of Equatorial Guinea located in the island of Bioko [15]. Nevertheless, CQ continued to be used in mainland region as the first-line treatment for uncomplicated malaria until 2009, and had been replaced by artesunate + sulphadoxine/pyrimethamine (AS+SP) combination on the island of Bioko in 2004 [16]. In 2009, artemisinin combination therapy (ACT) of artesunate/amodiaquine (AS/AQ) was adopted as first-line therapy based on the high levels of resistance to SP in neighbouring countries. More recently, a study conducted in Bata, the largest city in the mainland region, and Malabo revealed that AS/SP and AQ/SP combinations were both highly effective for the treatment of uncomplicated *P. falciparum* malaria [16]. SP is still prescribed alone for intermittent preventive therapy in pregnant women [17].

This study aimed to characterize the circulating populations of *Plasmodium* spp and *P. falciparum* from a combined set of human blood and mosquito samples collected in both coastal and inland villages from mainland Equatorial Guinea. *Plasmodium falciparum* diversity was analysed through the study of an antigen coding gene (*Pfmsp2*) as well as a set of neutral STR *loci* and four anti-malarial resistant associated genes (*Pfcrt*, *Pfmdr1*, *Pfdhfr* and *Pfdhps*). Finally, to trace the origin of anti-malarial resistance and its progression in this area, the presence of signatures of drug resistant selection in *P. falciparum* populations were investigated. The impact of these findings on control policies, especially the avoidance of dissemination of *P. falciparum* drug-resistant parasites in Equatorial Guinea, is discussed.

## Methods

### Sample collection

Peripheral blood samples from 97 inhabitants (zero to 78 years-old) were collected in 2005 in mainland Equatorial Guinea during the dry (February and August) and rainy (May) seasons from two villages, Miyobo (34 and 43 individuals in the dry and rainy seasons, respectively; 44 different individuals in total) and Ngonamanga (40 and 26 individuals in the dry and rainy seasons, respectively; 53 different individuals in total). Blood sampling has been performed in four consecutive days per individual, in order to better assess variations in the *P. falciparum* population’s composition. Further, 819 mosquito specimens were also collected during the same period and locations. Miyobo is located in a forested area on the riverbank of the Wele River, which crosses the mainland region from east to west. Ngonamanga is a coastal village surrounded by forest-savannah, 60 km north of Bata. In both, malaria is classified as hyperendemic. Both study areas and sample collection procedures have been described elsewhere [4].

Villagers were informed of the nature and aims of the study and voluntary participation of five households randomly selected by location was requested after approval of local authorities. Blood samples were collected after informed consent was received from all donors (parents or guardians responded on behalf of children). Mosquito collection was done after the approval of local authorities, the owner and occupants of the house. Written consent was obtained from the legal guardians of the recruited children and non-documented, oral consent was only requested from adults, due to the community-wide mistrust of signing official forms. The study was approved by the Ethical Committee of Equatorial Guinea’s Ministry of Health and Social Welfare, the National Malaria Control Programme, and local health authorities from the villages, which accepted the constraint and found no bio-ethical impediments to the study. Ethical clearance was also given by the Ethical Committees of the Instituto de Higiene e Medicina Tropical (Lisboa, Portugal) and the Instituto de Salud Carlos III (Madrid, Spain), according to EU regulations.

### DNA extraction and molecular assays

Individual mosquitoes, dried on silica gel, and blood spot samples were stored at room temperature until DNA preparation. DNA was extracted using chelex according to Plowe *et al*[18] from blood spots and to Arez *et al*[19] from mosquitoes.

Detection of malaria infection and identification of *Plasmodium* species was carried out by nested-PCR amplification of the ssrRNA genes [20]. *Plasmodium falciparum* positive samples were further genotyped for:

a) *Pfmsp2* gene by a nested-PCR as in Snounou *et al*[21];

b) Drug resistant associated genes by a nested PCR-RFLP analysis of the presence/absence of mutations at codons 75 and 76 of the *Pfcrt* gene, codons 86 and 1246 of the *Pfmdr1* gene, codons 51, 59, 108 and 164 of the *Pfdhfr* gene and codons 436, 437, 540 and 581 of the *Pfdhps* gene [22],

c) Nine neutral microsatellite *loci* (STR) distributed throughout the genome of *P. falciparum*: TAA42, TAA81 (chromosome 5), TA1, TAA87, TAA109 (chromosome 6), ARA2 (chromosome 11), TA102, PfPK2 and Pfg377 (chromosome 12). Primer sequences and PCR conditions are described in Anderson *et al*[23] and Conway *et al*[24];

d) STRs flanking *Pfdhfr and Pfdhps* genes located 0.3 kb, 4.4 kb and 5.3 kb upstream of codon 108 of *Pfdhfr* (chromosome 4) and 0.8 kb, 4.3 kb and 7.7 kb downstream from codon 437 of *Pfdhps* (chromosome 8). Primer sequences and PCR conditions are described in Roper *et al*[25], Ndiaye *et al*[26] and Salgueiro *et al*[27]. Southeast Asian *P. falciparum* K1 laboratory strain was used as reference (at STRs flanking the *Pfdhps* gene, the allelic composition of the K1 strain matches that of the East African *Pfdhps* double mutant A437G/K540E haplotype lineage SGE 1 [28]).

Amplified fragments were run in an automatic sequencer (ABI 3730, Applied Biosystem) at Yale University, DNA Analysis Facility on Science Hill. The software GeneMarker (SoftGenetics) was used to measure allele sizes. Samples that failed the amplification in any of the *loci* or presented multiple STR peaks were excluded for the haplotype definition [25]. A new haplotype was considered when there was one or more allelic changes across all *loci* considered. For the remaining analyses, in cases where multiple peaks were present, only the value of the highest peak per locus was scored [8].

### Statistical analysis

Pearson χ^2^ test was used to compare prevalence of infection between collection sites, seasons and hosts. Whenever Pearson χ^2^ test conditions were not satisfied, Fisher’s exact test was used (SPSS v.12 statistical software). Pearson’s χ^2^ test was also used to assess possible associations between *Plasmodium* species [29].

Prevalence of *Pfmsp2* alleles and the minimum number of concurrent genotypes in the same isolate (multiplicity of infection (MOI): the largest number of alleles found in each sample) were calculated for all comparison groups; mosquitoes *versus* blood samples, Miyobo *versus* Ngonamanga and rainy season *versus* dry season.

STR data was analysed with FSTAT v. 2.9.3.2 [30] to obtain measures of genetic diversity [allelic richness Rs: a measure of the number of alleles independent of sample size, hence allowing to compare this quantity between different sample sizes; and expected heterozygosity He per locus and sample: this use an unbiased estimator Hs, which is calculated from individual allele frequencies and range from zero (no heterozygosity) to nearly 1.0 (for a system with a large number of equally frequent alleles)] and genetic differentiation using the Fst estimator. Linkage disequilibrium (LD) tests were performed with GENEPOP v.3.4 [31].

After the assessment of PYR- and SFX-associated wild type (or sensitive) and mutant alleles, comparisons were made between populations classified as “wild type”, “single mutant” (*Pfdhfr*: 51 or 59 or 108 or 164; *Pfdhps:* 436 or 437 or 540 or 581), “double mutant” (*Pfdhfr:* 51:108 or 59:108 or 51:59 or 51:164; *Pfdhps*: 436:581 or 437:581 or 540:581), “triple mutant” (*Pfdhfr*: 51:59:108 or 59:108:164 or 51:59:164, *Pfdhps:* 436:437:581 or 436:540:581), “quadruple mutant” (*Pfdhfr*: 51:59:108:164, *Pfdhps* 436:437:540:581). However this was not always possible due to the low number of samples in some groups, so that only the whole sample was subdivided and compared according to geographic collection sites.

In multiple tests, Bonferroni correction was applied by dividing 0.05 by the number of tests to minimize type I errors and obtain the actual cut-off for significance [32].

## Results

### Detection and identification of *Plasmodium* species

A total of 427 blood samples from 97 individuals were collected in both villages and seasons (44 individuals from Miyobo and 53 from Ngonamanga). A total of 819 mosquitoes were collected (509 from Miyobo and 310 from Ngonamanga), 536 belonging to *Anopheles gambiae* complex, 259 belonging to *Anopheles nili* complex (presumably *Anopheles carnevalei*), three to *Anopheles funestus* complex and 21 *Anopheles moucheti moucheti*.

In order to determine prevalence of infection, an individual was defined as infected if he/she had at least one positive sample among the multiple samples collected; therefore, only one sample was considered per individual and all calculations were performed having the number of individuals as denominator. Overall, prevalence of *Plasmodium* spp infection in humans was 93% in Miyobo and 81% in Ngonamanga, and was higher in the dry season (69%) than in the rainy (67%). In mosquitoes, the prevalence of infection was slightly higher in Ngonamanga (20%) than Miyobo (19%) and in the rainy season (22%) than in the dry season (16%). Although the four *Plasmodium* species were detected in both hosts, *P. falciparum* was the predominant species occurring in 90% of the isolates (both humans and mosquitoes) either in single or mixed infection (see Additional files 1 and 2). In humans, *Plasmodium malariae* was the second most prevalent species, occurring in 13% of individuals, followed by *P. vivax* (10%) and finally *Plasmodium ovale* (8%) (see Additional file 1). In mosquitoes, *P. vivax* was the second most prevalent species (9%), followed by *P. malariae* (4%) and *P. ovale* (2%) (see Additional file 2). A significantly higher number than expected of mixed infections with *P. falciparum* and *P. malariae* in both hosts (blood samples: χ^2^=8.973, p=0.003; mosquitoes: χ^2^=15.745, p<0.001) was found. No association was found for the pair *P. falciparum* and *P. viv*ax.

### *Plasmodium falciparum* genetic diversity

#### Pfmsp2

*Plasmodium falciparum* was detected in 302 out of the 427 samples collected and successful genotyping of *Pfmsp2* gene was achieved in 73% (221/302) *P. falciparum*-positive blood samples and none in the 275 *P. falciparum*-positive mosquitoes. The unsuccessful amplification of *Pfmsp2* in mosquitoes was probably due to degradation of parasite DNA in dried mosquitoes stored at room temperature for a long period of time.

No major differences in allelic diversity were detected between seasons or villages, which shared 11 out of 13 alleles; two unique alleles were detected in Ngonamanga in dry season (IC_400 and IC_700) and only one was observed in the rainy season (FC27_250) in both villages. The mean MOI was slightly higher in Miyobo than in Ngonamanga; 1.98 *versus* 1.83, respectively, and varied between 1.46 (Ngonamanga, rainy season) and 2.19 (Ngonamanga, dry season). When values are compared between villages without season distinction, mean MOI was slightly higher in Miyobo than in Ngonamanga (1.98 *versus* 1.83, respectively) and it was higher in the dry season in Ngonamanga (dry *versus* rainy: 2.19 versus 1.46), while the opposite occurred in Miyobo (dry *versus* rainy: 1.88 *versus* 2.07).

#### Neutral STRs *loci*

Ninety-nine per cent (299/302) *P. falciparum*-positive blood samples and 83% (228/275) *P. falciparum*-positive mosquitoes were successfully genotyped. The number of observed alleles (Na), allelic richness (Rs) and genetic diversity (uH) are shown in Table 1. All nine STR analysed were polymorphic and the number of alleles varied between seven in Pfg377 and 17 in TA109 in human samples, and six in TA42 and 18 in TA109 in mosquitoes. The majority of samples presented multiple *P. falciparum* genotypes but in general, the most common alleles are shared between parasite populations present in humans and mosquitoes. Genetic diversity (uH) also presented similar values; 0.75 *versus* 0.77, in humans and mosquitoes, respectively, as well as the number of alleles for each *locus*; 12 *versus* 11, in humans and mosquitoes respectively (see Table 1).

**Table 1:** **Neutral microsatellite diversity of *Plasmodium falciparum *populations from Ngonamanga and Miyobo in humans and mosquitoes**

		**n**		**TA1**	**TA102**	**ARA2**	**TA87**	**Pfk2**	**TA81**	**TA42**	**Pfg377**	**TA109**	**Mean**
**Humans**	**TBs**	299	**Na**	13	12	9	15	14	11	14	7	17	12
			**Rs**	12	11	8	12	13	10	10	5	13	10
			**uH**	0.852	0.832	0.787	0.838	0.877	0.829	0.344	0.612	0.799	0.75
	**BsM**	195	**Na**	11	11	9	14	12	11	7	7	15	11
			**Rs**	10	9	8	9	10	9	5	5	9	8
			**uH**	0.846	0.848	0.771	0.829	0.857	0.792	0.293	0.642	0.811	0.74
	**BsN**	104	**Na**	11	8	8	9	11	10	11	5	9	9
			**Rs**	10	7	8	8	11	10	7	5	7	8
			**uH**	0.859	0.771	0.809	0.844	0.877	0.868	0.424	0.531	0.747	0.75
	**BsW**	141	**Na**	10	11	7	13	12	11	8	5	9	10
			**Rs**	9	10	7	10	11	10	6	5	8	8
			**uH**	0.819	0.854	0.797	0.840	0.874	0.851	0.307	0.634	0.805	0.753
	**BsD**	158	**Na**	11	10	9	11	12	10	11	7	13	10
			**Rs**	10	8	8	9	11	9	8	5	9	9
			**uH**	0.866	0.817	0.766	0.841	0.871	0.798	0.375	0.592	0.788	0.746
**Mosquitoes**	**TMq**	228	**Na**	13	12	8	11	12	10	6	8	18	11
			**Rs**	12	12	8	11	12	10	6	7	16	10
			**uH**	0.88	0.874	0.810	0.849	0.833	0.779	0.365	0.712	0.869	0.77
	**MqM**	130	**Na**	12	11	7	9	12	9	6	6	15	10
			**Rs**	10	11	7	8	10	8	6	5	13	9
			**uH**	0.851	0.870	0.780	0.845	0.845	0.753	0.462	0.713	0.848	0.77
	**MqN**	98	**Na**	9	7	7	8	8	8	3	6	14	8
			**Rs**	9	7	7	8	8	8	3	6	11	7
			**uH**	0.862	0.815	0.760	0.814	0.816	0.747	0.191	0.718	0.837	0.73
	**MqW**	86	**Na**	11	11	5	9	12	9	6	7	8	9
			**Rs**	10	11	5	9	11	9	6	6	8	8
			**uH**	0.882	0.904	0.741	0.823	0.846	0.774	0.428	0.717	0.808	0.769
	**MqD**	142	**Na**	10	7	8	9	10	8	4	5	17	9
			**Rs**	10	7	8	9	10	8	4	5	14	8
			**uH**	0.873	0.796	0.834	0.826	0.825	0.788	0.300	0.713	0.873	0.758

***n*: **sample size; ***TBs*: **total of blood samples; ***BsM*: **Blood samples from Miyobo; ***BsN*: **Blood samples from Ngonamanga; ***BsW*: **Blood samples wet season; ***BsD*: **Blood samples dry season; ***TMq*: **total mosquitoes; ***MqM*: **Mosquitoes from Miyobo; ***MqN*: **Mosquitoes from Ngonamanga; ***MqW*: **Mosquitoes wet season; ***MqD*: **Mosquitoes dry season; ***Na***: number of observed alleles; ***Rs***: allelic richness; ***uH*: **unbiased estimation of genetic diversity.

MOI varied between 1.62 (Miyobo, dry season, mosquitoes) and 2.25 (Ngonamanga, dry season, humans) and tended to be higher in humans than in mosquitoes (2.09 *versus* 1.80, respectively). When values are compared between villages without season distinction, mean MOI in humans was slightly higher in Ngonamanga (2.07) than in Miyobo (2.11), and conversely in mosquitoes; 1.69 and 1.91, Ngonamanga and Miyobo respectively. No significant genetic differentiation was observed among all study groups.

### Drug resistant associated genes

#### SNPs: *Pfcrt*, *Pfmdr1*, *Pfdhfr* and *Pfdhps*

**Figure 1:**
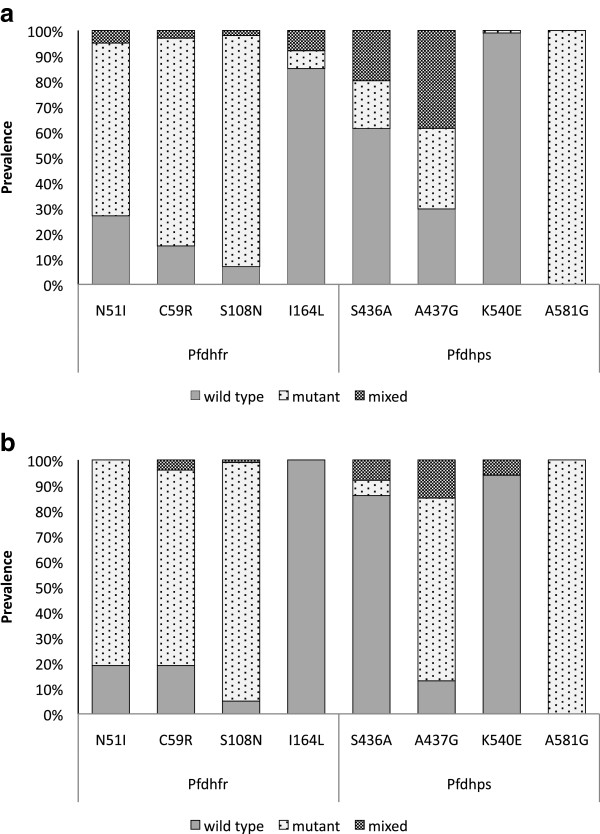
**Total prevalence of mutations in the eight codons of *Pfdhfr *and *Pfdhps *genes.**Legend: Prevalence of mutations in the *Pfdhfr *(N51I, C59R, S108N, I164L) and *Pfdhps *(S436A, A437G, K540, 581G), in single (wild type or mutant) and mixed infections in both seasons and localities in humans (**a**) and mosquitoes (**b**).

No major differences in the prevalence of mutant alleles were found among villages or seasons for any of the genes. Regarding *Pfcrt* and *Pfmdr1* genes associated with CQ resistance, the prevalence of the *Pfcrt* mutant alleles (N75E and K76T), present in single or mixed infection, was 56% and 72% in humans and 64% and 54% in mosquitoes; and a much higher prevalence of mutation in codon N86Y (84% and 61%, in humans and mosquitoes, respectively) than in D1246Y (1% in both hosts) was found in *Pfmdr1* gene (see Additional file 3). Regarding *Pfdhfr* gene, mutations N51I, C59R and S108N, associated with PYR resistance, presented prevalence, when in single or mixed infection, of 73%, 85%, 93% in humans and 81%, 81%, 95% in mosquitoes, respectively. The codon I164L was found in very low frequency (15% in humans and 0 in mosquitoes) (see Figure 1 and Additional file 3). While in Miyobo the double mutation (C59R/S108N) was the most prevalent in Ngonamanga most samples contained the triple mutation (N51I/C59R/S108N).

Mutations A437G and A581G in *Pfdhps* gene associated with SFX resistance were detected at very high prevalence, the latter reaching 100%, whereas a very low prevalence of codon K540E was found (1% in humans and 6% in mosquitoes only in mixed infections) (see Figure 1 and Additional file 3). Mutation S436A occurred in 38% in humans and 14% in mosquitoes. When comparing the two villages, no major differences were found in mosquitoes, and the prevalence of mutant alleles were: 12% in Ngonamanga and 20% in Miyobo, unlike in humans where the prevalence of this mutation was higher in Ngonamanga (51%) than in Miyobo (31%).

Most samples presented the double mutation (A437G/A581G), but many samples (approximately 38%) containing the triple mutation (A436/G437/G581) were identified in Ngonamanga.

Despite the high prevalence of resistance-associated mutations in *Pfdhfr* and *Pfdhps*, no parasites containing the quintuple mutation (N51I/C59R/S108N/A437G/K540E) associated with the clinical failure of SP combination were found.

#### STR *loci* flanking *Pfdhfr* and *Pfdhps* genes

The analysis using the STR flanking *Pfdhfr* and *Pfdhps* genes was only conducted in human isolates, since the amplification rate of these *loci* was very low in mosquito samples.

### Genetic diversity and linkage disequilibrium

The effect of SP selection on the *P. falciparum* population of Equatorial Guinea was evaluated by examining and comparing the levels of genetic diversity, LD and genetic differentiation between the *Pfdhfr* and *Pfdhps* flanking and neutral STR *loci*. Overall, genetic diversity estimated at neutral *loci* (*He* = 0.75; *Rs* = 14; *N* =244) was higher than at *loci* flanking both *Pfdhfr* (*He* = 0.15; *Rs* = 8; *N* =189) and *Pfdhps* (*He* = 0.80; *Rs* = 12; *N* = 189) genes (see Tables 2 and 3). In parasites holding *Pfdhfr* resistance associated alleles, triple mutants showed lower levels of genetic diversity (0.11) when compared to the single (0.36) and double mutants (0.21) (see Table 2). The mean *He* at three *Pfdhfr loci* was 0.22, which was much lower when compared to the mean *He* at 9 neutral *loci* (0.74) (see Table 2).

**Table 2:** **Statistics of the 15 STR loci of *Plasmodium falciparum*-positive individuals: mutants to PYR**

**Microsatellites**		**Sampled populations - Miyobo**										**Sampled populations - Ngonamanga**	
		**Single mutant**		**Double mutant**		**Triple mutant**		**All samples**				**Triple mutant**	
		**( *N *=10)**		**(N=37)**		**(N=17)**		**(N=64)**				**(N=82)**	
		***R***_***s***_	***H***_***e***_	***R***_***s***_	***H***_***e***_	***R***_***s***_	***H***_***e***_	***R***_***s***_	***H***_***e***_	***F***_***ST***_	***P***	***R***_***s***_	***H***_***e***_
***Loci *flanking *dhfr *gene**	*Dhfr *0.3	3	0.51	3	0.32	2	0.15	3	0.33	0.02	NS	7	0.26
	*Dhfr* 4.4	1	0.00	2	0.14	2	0.17	2	0.10	-0.04	NS	3	0.10
	*Dhfr* 5.3	3	0.56	2	0.18	1	0.00	2	0.24	0.10	0.03	3	0.08
	All *loci*	2	0.36	2	0.21	2	0.11	2	0.22	0.03	0.05	4	0.15
**Neutral *loci***	TA1	4	0.82	5	0.83	6	0.89	6	0.85	0.04	0.04	12	0.87
	TA102	4	0.64	7	0.88	5	0.84	6	0.79	0.03	NS	8	0.78
	ARA2	4	0.69	5	0.77	6	0.79	5	0.75	-0.03	NS	9	0.81
	TA87	4	0.87	6	0.85	5	0.81	6	0.84	0.01	NS	9	0.84
	PfPK2	5	0.93	6	0.86	5	0.85	6	0.88	-0.03	NS	12	0.87
	TA81	4	0.87	5	0.81	4	0.71	5	0.80	-0.04	NS	10	0.87
	TA42	3	0.60	2	0.11	2	0.28	2	0.32	0.06	NS	8	0.36
	Pfg377	3	0.67	3	0.56	3	0.65	3	0.62	-0.04	NS	7	0.55
	TA109	5	0.86	5	0.80	4	0.79	5	0.82	0.04	0.03	8	0.75
	All *loci*	4	0.77	5	0.72	4	0.73	5	0.74	<0.01	NS	9	0.74

***N*: **number of isolates genotyped, ***He*: **expected heterozygosity; ***Rs*: **allelic richness. **All *loci*: **mean over *loci Rs *and *He *and global *Fst* over *loci *as calculated by FSTAT. ***P:****P-*values of permutation tests to assess significance of *Fst* values. **NS: **non-significant (*P*>0.05).

**Table 3:** **Statistics of the 15 STR *loci *of *Plasmodium falciparum*-positive individuals: mutants to SFX**

**Microsatellites**		**Sampled populations - Miyobo**								**Sampled populations - Ngonamanga**							
		**Single mutant**		**Double mutant**		**All samples ( *N *=60)**				**Double mutant**		**Triple mutant**		**All samples (N=85)**			
										**(N=63)**		**(N=22)**					
		**(N=17)**		**( *N *=43)**													
		***R***_***s***_	***H***_***e***_	***R***_***s***_	***H***_***e***_	***R***_***s***_	***H***_***e***_	***F***_***ST***_	***P***	***R***_***s***_	***H***_***e***_	***R***_***s***_	***H***_***e***_	***R***_***s***_	***H***_***e***_	***F***_***ST***_	***P***
***Loci *flanking *dhps *gene**	*Dhps* 0.8	4	0.60	5	0.75	5	0.67	0.02	NS	9	0.81	5	0.64	9	0.73	0.06	0.01
	*Dhps *4.3	6	0.83	5	0.77	6	0.80	<0.01	NS	7	0.78	4	0.71	7	0.75	0.13	<0.01
	*Dhps *7.7	6	0.79	7	0.84	8	0.82	0.09	<0.01	10	0.85	6	0.67	10	0.76	0.14	<0.01
	All *loci*	5	0.74	6	0.79	6	0.76	0.04	<0.01	9	0.81	5	0.67	9	0.74	0.11	<0.01
**Neutral *loci***	TA1	5	0.79	8	0.87	8	0.83	0.04	NS	9	086	8	0.88	9	0.87	<0.01	NS
	TA102	5	0.78	9	0.87	9	0.83	0.04	0.01	6	0.75	7	0.86	7	0.80	<-0.01	NS
	ARA2	6	0.79	6	0.69	7	0.74	0.06	<0.01	7	0.80	6	0.82	7	0.81	-0.01	NS
	TA87	5	0.79	8	0.84	7	0.82	0.05	0.02	8	0.83	7	0.87	8	0.85	0.02	NS
	PfPK2	5	0.83	8	0.80	8	0.81	0.11	0.02	10	0.87	8	0.87	11	0.87	0.02	<0.05
	TA81	5	0.77	6	0.78	7	0.78	-0.02	NS	9	0.88	8	0.83	9	0.85	<0.01	NS
	TA42	1	0.00	4	0.29	3	0.14	0.03	NS	5	0.44	3	0.18	5	0.31	0.05	0.04
	Pfg377	3	0.56	4	0.59	3	0.57	<-0.01	NS	5	0.59	3	0.40	5	0.50	0.01	NS
	TA109	6	0.86	6	0.81	6	0.83	-0.02	NS	5	0.72	5	0.60	6	0.66	0.16	<0.01
	All *loci*	5	0.69	6	0.73	6	0.71	0.03	<0.01	7	0.75	6	0.70	7	0.72	0.03	0.01

***N*: **number of isolates genotyped, ***He*: **expected heterozygosity; ***Rs*: **allelic richness. **All *loci*: **mean over *loci Rs *and *He *and global *Fst* over *loci *as calculated by FSTAT. ***P:****P-*values of permutation tests to assess significance of *Fst* values. **NS: **non-significant (*P*>0.05).

The reduction in the genetic diversity is not so marked in *Pfdhps* as in *Pfdhfr*. When double mutants and triple mutants are compared, there is a slight decrease in genetic diversity (*He*=0.81 for double *versus He*=0.67 for triple mutants) but values are still similar and high (see Table 3). Statistical tests for LD were conducted for all pairs of flanking STR on each of the mutant groups – single, double and triple mutants (105 possible tests for *Pfdhfr* and 316 for *Pfdhps*). Only two associations showed significant results (p <0.05), after Bonferroni’s correction was applied, in the *Pfdhps* double mutants group, involving *loci* 0.8 kb/4.3 kb and 4.3 kb/7.7 kb. No significant pairwise association was found involving the *Pfdhfr* gene.

### *Pfdhfr* and *Pfdhps* haplotype characterization

Only samples with single infections and successful amplification of all *loci* were used for the haplotype characterization. Thus, haplotypes were reconstructed in 57 out of 298 human isolates genotyped for *Pfdhfr* and in 35 out of 296 human isolates genotyped for *Pfdhps*. For the *Pfdhfr* gene, nine distinct haplotypes were found (see Additional file 4). The haplotype H9, an exact match of the *P. falciparum* K1 strain used as a control (double mutation C59R/S108N and allele sizes of 113 bp, 183 bp and 210 bp to the 0.3 kb, 4.4 kb and 5.3 kb *loci*, respectively), was found in 10 samples from Miyobo. Most frequent haplotypes in 53 out of 57 samples (H1, H3, H5, H8) also matched STR sizes in K1 strain.

The majority of samples from Ngonamanga (97%) showed the triple-mutant IRNI (51I:59R:108N:164I – mutated codons appear underlined), while in Miyobo the most prevalent haplotype was the double-mutant NRNI (51N: 59R:108N:164I) (43%) followed by the triple-mutant IRNI with 26% (see Additional file 4).

Regarding the *Pfdhps* gene, 25 distinct haplotypes were found (see Additional file 5); 16 in Miyobo and nine in Ngonamanga, only one shared between the two villages. None of the haplotypes found matches with K1 strain (single mutation A437G and allele sizes of 131 bp, 103 bp and 108 bp to the 0.8 kb, 4.3 kb and 7.7 kb *loci*, respectively). The haplotypes found for the *Pfdhps* gene have multiple independent lineages since the majority of the haplotypes were unique. Nevertheless, the most prevalent haplotype in Ngonamanga was the triple-mutant AGKG (436A:437G:540K:581G) with 38%, whilst in Miyobo it was the double-mutant SGKG (436S:437G:540 K:581G) (63%).

## Discussion

Malaria still is a major public health concern in Equatorial Guinea, especially in the mainland. In order to contribute to the update of the malaria situation in this area, a combined set of blood and mosquito samples from the same locations were analysed to characterize the genetic diversity of circulating populations of *Plasmodium* spp and especially of *P. falciparum*, in both hosts.

### *Plasmodium* species diversity

This study presents a much higher prevalence of *Plasmodium* infection in mainland Equatorial Guinea (87%) than the one reported for the Insular Region in 2005 (32%) [33]. This difference is likely due to the fact that most malaria control activities have been deployed on the island of Bioko where the capital, Malabo is located. In 2004, the first stage of the project “The Bioko Island Malaria Control Project (BIMCP)” was launched and initial reports stated a significant decrease in the prevalence of infection, achieving an overall malaria prevalence of 18% in 2008 [10,34]. In the present study, although *P. falciparum* infections were the most frequent, *P. vivax* infections were detected for the first time both in humans and mosquitoes, which means that active transmission of this species not previously reported in this area is occurring. The apparent higher presence of *P. vivax* in mosquitoes might be due to its higher visibility in the vector, since in the human host this parasite can form dormant forms in the liver – hypnozoites - and go unnoticed, as discussed in [4].

Regarding mixed infections, *P. falciparum* and *P. malariae* are also associated in mainland Equatorial Guinea, as has been reported in other sub-Saharan countries [3,35-39]. This association was observed both in humans and mosquitoes, which suggests that no differing patterns of *Plasmodium* species association in the two hosts occurs as has formerly been reported in Guinea Bissau [3].

### *Plasmodium falciparum* genetic diversity

Concerning *P. falciparum* genetic diversity, the analysis of both *Pfmsp2* and neutral STR in humans showed similar levels of allelic diversity and MOI in both villages and seasons. No reduction of genotype diversity or MOI was observed with the decline of transmission, as seen in areas of lower endemicity, such as Sudan [40] or in areas with marked differences in malaria endemicity [41]. However, analogous results were obtained by Cano *et al*[42] in a study conducted on the island of Annobon, part of Equatorial Guinea Insular Region.

In mosquitoes, this analysis was only possible with the neutral STR and the results confirmed those obtained in humans, i. e., high levels of genetic diversity and no significant genetic differentiation between geographic locations, despite their different ecological differences or seasons. This is a sign of high malaria endemicity in mainland Equatorial Guinea and the similarity between population genetic structures is concordant with other studies in African highly malaria-endemic countries [8,40]. No significant genetic differentiation was seen between hosts, when comparisons between human blood samples and mosquitoes were made using neutral STR data. The most common alleles are found in both humans and mosquitoes, which may indicate consistency in the parasite populations that are being transmitted. Nevertheless, MOI values were higher in humans than in mosquitoes. As Arez *et al*[3] observed, a higher proportion of single-genotype infections in mosquitoes could point to a limited genetic diversity of the *inocula* and a high genetic diversity in humans resulting from superinfection phenomena.

### Anti-malarial resistance evolution

The prevalence of the main point mutations associated with CQ resistance (75E and 76 T of *Pfcrt* gene and 86Y of *Pfmdr1* gene) was nearly 71%. Although the mutation 1246Y in the *Pfmdr1* gene has also been associated with reduced susceptibility to CQ [43], a very low frequency of this mutation was found in Equatorial Guinea (1%).

Nowadays, after the introduction of artemisinin-based combination therapy (ACT), decrease in prevalence of mutations associated with CQ resistance might be expected, due to the absence of drug pressure, as reported in Malawi, China, Kenya and Angola [44-47]. However, a recent study conducted in Equatorial Guinea [48] found higher prevalence of mutation in *Pfcrt* (codon 76) and in *Pfmdr1* (codon 1246) (98% and 96%, respectively), than those found in this study (72% for *Pfcrt* codon 76 and 1% for *Pfmdr1* codon 1246) in isolates collected in 2005, when CQ was still in use in mainland Equatorial Guinea. The increasing of these and other point mutants might be a result of selective pressure by AS-AQ combination, since AQ is a close Mannich base analogue of CQ, promoting the maintenance of CQ-resistant isolates with the mutant *Pfcrt* and *Pfmdr1* genotypes. On the other hand, another possibility is the continuous use of CQ despite national therapeutic guidelines [49].

In Equatorial Guinea, SP has been used as a second-line therapy for many years and lately, though less intensely, as a first-line in combination with artemisinin derivatives and it is used in preventive chemotherapy in pregnancy. Although the failure rate of this combination has not suffered major variations since 1992, and in the late 1990s was still 10% [15], it was expected that the continuous use of this drug would rapidly lead to an increase of resistance levels as had happened in other countries such as Kenya [50] and Tanzania [51].

In fact, a high prevalence of mutation in genes associated with resistance to the SP combination (~70%) was observed in this study. PYR resistance seemed to be well established in mainland Equatorial Guinea and nearly 80% of parasite populations presented the triple mutant N51I/C59R/S108N in the *Pfdhfr* gene, both in humans and mosquitoes, as seen in other nearby countries such as Cameroon [52], Gabon [53] and São Tomé and Principe [27].

Regarding SFX resistance, a high prevalence of the mutation A437G in *Pfdhps* was detected. However the mutation K540E was practically non-existent, which is usual in West Africa [28]. The prevalence of S436A mutation was low, contrary to data from the neighbouring country Gabon, where this was the most frequent *Pfdhps* polymorphism [54]. The mutations S436A and A581G are less studied due to their low prevalence in some African countries, and the lack of knowledge of their role in treatment failure [55]. However, the prevalence of A581G mutation in this study reached 100%. Other recent studies conducted in different African countries showed an increase of the prevalence of A581G, during the last years [53,56].

The quintuple mutant, associated with SP clinical failure [57,58] and resulting from the combination of the *Pfdhfr* triple mutant N51I/C59R/S108N (linked to resistance to PYR) with the *Pfdhps* double mutant A437G/K540E (linked to resistance to SFX), was not detected since no samples containing the latter were found. No major differences in the prevalence of mutation between parasites in humans and mosquitoes occurred.

Analysis on *Pfdhfr* flanking STRs showed that the majority of haplotypes found were associated with triple mutants IRNI, especially in Ngonamanga, while the majority of isolates harboured double mutants NRNI in Miyobo. These two haplotypes have already been reported in Ghana [59] and the triple mutant IRNI was also found in Southeast Asia [60]. The majority of the haplotypes seems to have a single origin. In fact, the haplotypes found were very similar among them, with the majority of them corresponding to the H3 haplotype. This haplotype has arisen from H9 haplotype double mutant through an additional mutation occurring at position 59 of the *Pfdhfr* gene. Both H3 and H9 haplotypes share the same microsatellite profile.

The results of the present study suggest that PYR resistance was firstly established in Ngonamanga, probably due to the fact that Miyobo is more isolated and the introduction of the drug may have occurred later. It is likely that SP combination has been introduced first in Malabo (the capital of the country), and then its utilization was spread all over the country. Ngonamanga, being a coastal area (closer to the capital), may have started to use this drug earlier, and therefore to develop resistance sooner. The process of the addition of a single mutation in *Pfdhfr* alleles to double mutants, originating a high prevalence of triple mutants [61] was still occurring in Miyobo. The most common haplotype 113/183/210 with the triple mutant IRNI, already described in Senegal [26], should be related to the 109/183/210 background, found in Tanzania, South Africa, Southeast Africa [25] and, most recently, in Kenya [50].

It was expected that the extensive use of SP would lead to a rapid increase in resistance levels, leaving signatures of drug selective pressure, such as a reduction in genetic diversity around *Pfdhps* and *Pfdhfr* due to selective sweep; an increased genetic differentiation at the *loci* under selection; and, a significant LD between *loci* flanking *Pfdhps* and *Pfdhfr* genes [62].

Indeed, the reduction in heterozigosity in the *loci* flanking *Pfdhfr* gene with regard to the mean of heterozigosity in the neutral *loci* indicates that this gene has undergone strong selection in Equatorial Guinea. The higher mean of *He* around double mutant than the mean of *He* around triple mutant is consistent with a model of positive directional selection. The Fst values at STR *loci* linked to *Pfdhfr* gene were higher when compared with mean Fst at neutral *loci*, which supports this hypothesis. However, no significant LD values were found between flanking genes of interest.

According to the results herein presented, SFX resistance seems to have appeared more recently than PYR resistance in mainland Equatorial Guinea. Indeed, only mutations at codons A437G and A581G, from the *Pfdhps* polymorphic sites surveyed showed high prevalence. Point mutations at S436A and K540E codons were rarely seen. A wide diversity of haplotypes was detected, being the majority unique haplotypes, which is consistent with independent origins for those alleles. The most prevalent haplotype match with AGK1/SGK1 lineages of West African origin and a few others (436A:437G:540K/ 436S:437G:540 K) with probable independent origin. The double mutant lineage identified as SGE1 (436S:437G:540E), originated in East Africa [28], was not detected in this study. As occurred with PYR resistance, the resistance to SFX seems to have been established earlier in Ngonamanga, where the prevalence of triple mutants is higher than in Miyobo.

When double mutants (*He*=0.81) were compared with triple mutants (*He*=0.67), a reduction in the heterozigosity was seen. However the values found are remarkably higher when comparing to those found for the PYR resistance (mean *He*=0.22). These differences may be due to the presence of multiple lineages occurring within individual populations. Also, significant LD values were found between flanking *Pfdhps* gene, involving *loci* 0.8 kb/4.3 kb and 4.3 kb/7.7Kb. Overall, these results might be suggestive of soft selective sweep, where multiple lineages are superimposed within a single population causing higher *He* values than in populations where a single lineage is present [63].

The results gathered in this study suggests that the PYR resistance has been established for a while in mainland Equatorial Guinea leaving selection signatures as the decrease in genetic diversity and an increased genetic differentiation at the *loci* around *Pfdhfr* gene. In addition, the impact on genetic diversity was less clear at the *loci* flanking *Pfdhps*, with only evidence of a soft selective sweep effect. This agrees with a more recent introduction of resistance to SFX in Equatorial Guinea, which is in agreement with results obtained in a recent study [64].

## Conclusions

CQ is no longer recommended for malaria treatment in Equatorial Guinea but SP remains in use in combination with artesunate and is the only drug recommended for intermittent preventive therapy in pregnancy [65]. Prevalence of infection in the mainland region, where most of the country’s population live, remains high despite the efforts undertaken to control malaria transmission mainly on the island of Bioko [10,34]. A close and continuous monitoring of point mutations frequency in the two genes associated with SP resistance, *Pfdhfr* and *Pfdhps*, is essential since there is the danger of an eventual reduction in the efficacy of SP combined therapy.

## Competing interests

The authors declare that they have no competing interests.

## Authors’ contributions

CM and VG carried out the laboratory analysis. JC carried out the sampling and field data collection. PS, PB, BdS, VER, AB and JC participated in the analysis and interpretation of data and helped to draft the manuscript. CM and APA drafted the paper. APA designed the study and participated in the analysis and interpretation of data. All authors read and approved the final manuscript.

## Supplementary Material

Additional file 1 **Prevalence of *Plasmodium *infections in humans, in two villages of mainland Equatorial Guinea.** n: sample size; F: *P. falciparum*; M: *P. malariae*; O: *P. ovale;* V: *P. vivax*; F+M: mixed infection by *P. falciparum *and *P. malariae;* F+O: mixed infection by *P. falciparum *and *P. ovale*; F+V: mixed infection by *P. falciparum *and *P. vivax; *F+M+O: mixed infection by *P. falciparum*, *P. malariae *and *P. ovale*; F+M+V: mixed infection by *P. falciparum*, *P. malariae *and *P. vivax*. Click here for file

Additional file 2 **Prevalence of *Plasmodium *infections in mosquitoes, in two villages of mainland Equatorial Guinea.** n: sample size; F: *P. falciparum*; M: *P. malariae*; O: *P. ovale;* V: *P. vivax*; F+M: mixed infection by *P. falciparum* and *P. malariae;* F+O: mixed infection by *P. falciparum* and *P. ovale*; F+V: mixed infection by *P. falciparum *and *P. vivax; *F+M+O: mixed infection by *P. falciparum*, *P. malariae *and *P. ovale*; F+M+V: mixed infection by *P. falciparum*, *P. malariae *and *P. vivax*. Click here for file

Additional file 3 **Characterization of mutations in *Pfcrt, Pfmdr1*, *Pfdhps *and *Pfdhfr *genes, in humans and mosquitoes.** Click here for file

Additional file 4 ***Pfdhfr *point mutations and their respective STR haplotypes in allele size.** Click here for file

Additional file 5 ***Pfdhps *point mutations and their respective STR haplotypes in allele size.** Click here for file
