# Supplementary material for: Genetic diversity and signatures of selection of drug resistance in Plasmodium populations from both human and mosquito hosts in continental Equatorial Guinea
Source: Malar J. 2013 Mar 27;12:114. doi: 10.1186/1475-2875-12-114 (PMC3621214; doi:10.1186/1475-2875-12-114)
Supplement: Additional file 1 — Prevalence of Plasmodium infections in humans, in two villages of mainland Equatorial Guinea. n: sample size; F: P. falciparum; M: P. malariae; O: P. ovale; V: P. vivax; F+M: mixed infection by P. falciparum and P. malariae; F+O: mixed infection by P. falciparum and P. ovale; F+V: mixed infection by P. falciparum and P. vivax; F+M+O: mixed infection by P. falciparum, P. malariae and P. ovale; F+M+V: mixed infection by P. falciparum, P. malariae and P. vivax. [file 1475-2875-12-114-S1.docx]

|  | **Village** | **Ngonamanga** | | | **Miyobo** | | | **Total** | |
| --- | --- | --- | --- | --- | --- | --- | --- | --- | --- |
|  | **Season** | **Dry** | **Rainy** | **Total** | **Dry** | **Rainy** | **Total** | **n** | **%** |
|  | **n** | 40 | 26 | 53 | 34 | 43 | 44 | **97** | **-** |
|  | **n of positive samples** | 26 (65%) | 17 (65.4%) | 43 (81.1%) | 25 (73.5%) | 30 (68.8%) | 41 (93.2%) | **84** | **86.6** |
| **Single infection** | **F** | 24 | 15 | 68 | 18 | 17 | 52 | 62 | 63.9 |
|  | **M** | 0 | 0 | 0 | 2 | 0 | 4 | 2 | 2.1 |
|  | **O** | 0 | 0 | 0 | 0 | 0 | 0 | 0 | 0.0 |
|  | **V** | 0 | 0 | 0 | 2 | 0 | 4 | 2 | 2.1 |
| **Mixed infection** | **F + M** | 2 | 0 | 4 | 1 | 3 | 7 | 5 | 5.2 |
|  | **F + O** | 0 | 2 | 4 | 2 | 2 | 4 | 4 | 4.1 |
|  | **F + V** | 0 | 0 | 0 | 2 | 2 | 11 | 5 | 5.2 |
|  | **F + M + O** | 0 | 0 | 0 | 0 | 3 | 7 | 3 | 3.1 |
|  | **F + M + V** | 0 | 0 | 0 | 0 | 1 | 2 | 1 | 1.0 |
|  | **Total** | 2 | 2 | 8 | 5 | 11 | 32 | 18 | 18.6 |
| **Overall infection F** | | **26** | **17** | **100** | **23** | **28** | **90** | **80** | **95.2** |
| **Overall infection M** | | **2** | **0** | **5** | **3** | **7** | **22** | **11** | **13.1** |
| **Overall infection V** | | **0** | **0** | **0** | **4** | **3** | **20** | **8** | **9.5** |
| **Overall infection O** | | **0** | **2** | **5** | **2** | **5** | **12** | **7** | **8.3** |

**Additional file 1.** Prevalence of *Plasmodium* infections in humans, in two villages of mainland Equatorial Guinea.

**n:** sample size; **F:** *P. falciparum*; **M:** *P. malariae*; **O:** *P. ovale;* **V:** *P. vivax*; **F+M:** mixed infection by *P. falciparum* and *P. malariae;* **F+O:** mixed infection by *P. falciparum* and *P. ovale*; **F+V:** mixed infection by *P. falciparum* and *P. vivax;* **F+M+O:** mixed infection by *P. falciparum*, *P. malariae* and *P. ovale*; **F+M+V:** mixed infection by *P. falciparum*, *P. malariae* and *P. vivax.*
