# Supplementary material for: Genetic diversity and signatures of selection of drug resistance in Plasmodium populations from both human and mosquito hosts in continental Equatorial Guinea
Source: Malar J. 2013 Mar 27;12:114. doi: 10.1186/1475-2875-12-114 (PMC3621214; doi:10.1186/1475-2875-12-114)
Supplement: Additional file 2 — Prevalence of Plasmodium infections in mosquitoes, in two villages of mainland Equatorial Guinea. n: sample size; F: P. falciparum; M: P. malariae; O: P. ovale; V: P. vivax; F+M: mixed infection by P. falciparum and P. malariae; F+O: mixed infection by P. falciparum and P. ovale; F+V: mixed infection by P. falciparum and P. vivax; F+M+O: mixed infection by P. falciparum, P. malariae and P. ovale; F+M+V: mixed infection by P. falciparum, P. malariae and P. vivax. [file 1475-2875-12-114-S2.docx]

**Additional file 2.** Prevalence of *Plasmodium* infections in mosquitoes, in two villages of mainland Equatorial Guinea.

| **Village** | | **Ngonamanga** | | | | **Miyobo** | | | | **Total** | |
| --- | --- | --- | --- | --- | --- | --- | --- | --- | --- | --- | --- |
| **Season** | | **Dry** | | **Rainy** | | **Dry** | | **Rainy** | |  |  |
|  | | **Head +Torax** | **Abdomen** | **Head +Torax** | **Abdomen** | **Head +Torax** | **Abdómen** | **Head +Torax** | **Abdomen** | **n** | **%** |
| **n** | | 249 | 249 | 61 | 61 | 386 | 386 | 123 | 123 | 1638 | % |
| **n of positive samples** | | 46 | 54 | 9 | 14 | 31 | 55 | 24 | 42 | **275** | **16.8** |
|  |  | **18.50%** | **21.60%** | **14.80%** | **23%** | **8.03%** | **14.25%** | **19.51%** | **34.15%** |  |  |
| **Single infection** | **F** | 39 | 47 | 7 | 14 | 29 | 42 | 24 | 35 | 237 | 14.5 |
|  | **V** | 4 | 4 | 2 | 0 | 2 | 10 | 0 | 0 | 22 | 1.3 |
|  | **M** | 1 | 0 | 0 | 0 | 0 | 2 | 0 | 1 | 4 | 0.2 |
|  | **O** | 0 | 0 | 0 | 0 | 0 | 0 | 0 | 0 | 0 | 0.0 |
| **Mixed infection** | **F + V** | 1 | 0 | 0 | 0 | 0 | 1 | 0 | 0 | 2 | 0.1 |
|  | **F + M** | 0 | 2 | 0 | 0 | 0 | 0 | 0 | 4 | 6 | 0.4 |
|  | **F + O** | 0 | 1 | 0 | 0 | 0 | 0 | 0 | 2 | 3 | 0.2 |
|  | **F + M + O** | 1 | 0 | 0 | 0 | 0 | 0 | 0 | 0 | 1 | 0.1 |
| **Overall infection F** | | **41** | **50** | **7** | **14** | **29** | **43** | **24** | **41** | **249** | **90.5** |
| **Overall infection V** | | **5** | **4** | **2** | **0** | **2** | **11** | **0** | **0** | **24** | **8.7** |
| **Overall infection M** | | **2** | **2** | **0** | **0** | **0** | **2** | **0** | **5** | **11** | **4.0** |
| **Overall infection O** | | **1** | **1** | **0** | **0** | **0** | **0** | **0** | **2** | **4** | **1.5** |

**n:** sample size; **F:** *P. falciparum*; **M:** *P. malariae*; **O:** *P. ovale;* **V:** *P. vivax*; **F+M:** mixed infection by *P. falciparum* and *P. malariae;* **F+O:** mixed infection by *P. falciparum* and *P. ovale*; **F+V:** mixed infection by *P. falciparum* and *P. vivax;* **F+M+O:** mixed infection by *P. falciparum*. *P. malariae* and *P. ovale*; **F+M+V:** mixed infection by *P. falciparum*. *P. malariae* and *P. vivax*
