# Supplementary material for: Genetic diversity and signatures of selection of drug resistance in Plasmodium populations from both human and mosquito hosts in continental Equatorial Guinea
Source: Malar J. 2013 Mar 27;12:114. doi: 10.1186/1475-2875-12-114 (PMC3621214; doi:10.1186/1475-2875-12-114)
Supplement: Additional file 3 — Characterization of mutations in Pfcrt, Pfmdr1, Pfdhps and Pfdhfr genes, in humans and mosquitoes. [file 1475-2875-12-114-S3.docx]

**Additional file 3.** Characterization of mutations in *Pfcrt*, *Pfmdr1*, *Pfdhps* and *Pfdhfr* genes, in humans and mosquitoes.

|  | | | ***Pfcrt*** | | ***Pfmdr1*** | | ***Pfdhfr*** | | | | ***Pfdhps*** | | | |
| --- | --- | --- | --- | --- | --- | --- | --- | --- | --- | --- | --- | --- | --- | --- |
|  |  |  | **N75E** | **K76T** | **N86Y** | **D1246Y** | **N51I** | **C59R** | **S108N** | **I164L** | **S436A** | **A437G** | **K540E** | **A581G** |
| **Humans** | **Ngonamanga** | **n** | 113 | 113 | 113 | 113 | 113 | 113 | 113 | 113 | 113 | 113 | 113 | 113 |
|  |  | **wild type** | 0.25 | 0.22 | 0.15 | 1.0 | 0.02 | 0.02 | 0.02 | 0.98 | 0.49 | 0.30 | 0.98 | 0.00 |
|  |  | **mutant** | 0.67 | 0.58 | 0.56 | 0.0 | 0.91 | 0.92 | 0.95 | 0.02 | 0.22 | 0.26 | 0.02 | 1.00 |
|  |  | **mixed** | 0.08 | 0.20 | 0.29 | 0.0 | 0.07 | 0.06 | 0.04 | 0.00 | 0.29 | 0.44 | 0.00 | 0.00 |
|  | **Miyobo** | **n** | 189 | 184 | 181 | 180 | 189 | 189 | 189 | 189 | 186 | 184 | 189 | 189 |
|  |  | **wild type** | 0.56 | 0.31 | 0.16 | 0.98 | 0.42 | 0.23 | 0.10 | 0.77 | 0.69 | 0.29 | 1.00 | 0.00 |
|  |  | **mutant** | 0.44 | 0.54 | 0.65 | 0.00 | 0.54 | 0.75 | 0.88 | 0.11 | 0.17 | 0.35 | 0.00 | 1.00 |
|  |  | **mixed** | 0.15 | 0.15 | 0.19 | 0.02 | 0.04 | 0.02 | 0.02 | 0.12 | 0.14 | 0.35 | 0.00 | 0.00 |
|  | **Dry season** | **n** | 168 | 166 | 167 | 166 | 166 | 166 | 166 | 166 | 166 | 167 | 166 | 166 |
|  |  | **wild type** | 0.43 | 0.20 | 0.16 | 0.99 | 0.13 | 0.10 | 0.01 | 0.91 | 0.54 | 0.25 | 1.00 | 0.00 |
|  |  | **mutant** | 0.54 | 0.66 | 0.62 | 0.00 | 0.81 | 0.87 | 0.96 | 0.01 | 0.17 | 0.30 | 0.00 | 1.00 |
|  |  | **mixed** | 0.02 | 0.14 | 0.22 | 0.01 | 0.07 | 0.04 | 0.03 | 0.08 | 0.30 | 0.46 | 0.00 | 0.00 |
|  | **Wet season** | **n** | 134 | 131 | 127 | 127 | 136 | 136 | 136 | 136 | 133 | 130 | 136 | 136 |
|  |  | **wild type** | 0.45 | 0.37 | 0.16 | 0.98 | 0.45 | 0.22 | 0.14 | 0.78 | 0.71 | 0.36 | 0.99 | 0.00 |
|  |  | **mutant** | 0.51 | 0.41 | 0.61 | 0.00 | 0.52 | 0.75 | 0.85 | 0.15 | 0.01 | 0.34 | 0.01 | 1.00 |
|  |  | **mixed** | 0.04 | 0.21 | 0.24 | 0.02 | 0.03 | 0.03 | 0.01 | 0.00 | 0.08 | 0.30 | 0.00 | 0.00 |
|  | **Total** | **n** | 302 | 297 | 294 | 293 | 302 | 302 | 302 | 302 | 299 | 297 | 302 | 302 |
|  |  | **wild type** | 0.44 | 0.28 | 0.16 | 0.99 | 0.27 | 0.15 | 0.07 | 0.85 | 0.62 | 0.30 | 0.99 | 0 |
|  |  | **mutant** | 0.53 | 0.55 | 0.62 | 0.00 | 0.68 | 0.81 | 0.91 | 0.07 | 0.19 | 0.32 | 0.01 | 1 |
|  |  | **mixed** | 0.03 | 0.17 | 0.23 | 0.01 | 0.05 | 0.03 | 0.02 | 0.08 | 0.20 | 0.39 | 0.00 | 0 |
| **Mosquitoes** | **Ngonamanga** | **n** | 30 | 27 | 23 | 25 | 36 | 36 | 32 | 32 | 28 | 27 | 30 | 30 |
|  |  | **wild type** | 0.13 | 0.1 | 0.65 | 0.96 | 0.25 | 0.25 | 0.00 | 1.00 | 0.89 | 0.15 | 0.90 | 0.00 |
|  |  | **mutant** | 0.83 | 0.6 | 0.26 | 0.04 | 0.75 | 0.69 | 1.00 | 0.00 | 0.11 | 0.78 | 0.00 | 1.00 |
|  |  | **mixed** | 0.03 | 0.2 | 0.09 | 0.00 | 0.00 | 0.06 | 0.00 | 0.00 | 0.00 | 0.07 | 0.10 | 0.00 |
|  | **Miyobo** | **n** | 28 | 38 | 39 | 53 | 17 | 17 | 51 | 51 | 49 | 48 | 54 | 43 |
|  |  | **wild type** | 0.61 | 0.68 | 0.23 | 1.00 | 0.06 | 0.06 | 0.08 | 1.00 | 0.84 | 0.13 | 0.96 | 0.00 |
|  |  | **mutant** | 0.32 | 0.18 | 0.72 | 0.00 | 0.94 | 0.94 | 0.90 | 0.00 | 0.04 | 0.69 | 0.00 | 1.00 |
|  |  | **mixed** | 0.07 | 0.13 | 0.05 | 0.00 | 0.00 | 0.00 | 0.02 | 0.00 | 0.12 | 0.19 | 0.04 | 0.00 |
|  | **Dry season** | **n** | 34 | 24 | 25 | 33 | 34 | 34 | 31 | 31 | 34 | 36 | 37 | 37 |
|  |  | **wild type** | 0.21 | 0.42 | 0.32 | 0.97 | 0.26 | 0.24 | 0.10 | 1.00 | 0.91 | 0.08 | 0.95 | 0.00 |
|  |  | **mutant** | 0.74 | 0.5 | 0.60 | 0.03 | 0.74 | 0.74 | 0.90 | 0.00 | 0.06 | 0.83 | 0.00 | 1.00 |
|  |  | **mixed** | 0.06 | 0.08 | 0.08 | 0.00 | 0.00 | 0.03 | 0.00 | 0.00 | 0.03 | 0.08 | 0.05 | 0.00 |
|  | **Wet season** | **n** | 24 | 41 | 37 | 45 | 19 | 19 | 52 | 52 | 43 | 39 | 47 | 36 |
|  |  | **wild type** | 0.58 | 0.49 | 0.43 | 1.00 | 0.05 | 0.11 | 0.02 | 1.00 | 0.81 | 0.18 | 0.94 | 0.00 |
|  |  | **mutant** | 0.38 | 0.29 | 0.51 | 0.00 | 0.95 | 0.84 | 0.96 | 0.00 | 0.07 | 0.62 | 0.00 | 1.00 |
|  |  | **mixed** | 0.04 | 0.22 | 0.05 | 0.00 | 0.00 | 0.05 | 0.02 | 0.00 | 0.12 | 0.21 | 0.06 | 0.00 |
|  | **Total** | **n** | 58 | 65 | 62 | 78 | 53 | 53 | 83 | 83 | 77 | 75 | 84 | 73 |
|  |  | **wild type** | 0.36 | 0.46 | 0.39 | 0.99 | 0.19 | 0.19 | 0.05 | 1.00 | 0.86 | 0.13 | 0.94 | 0.00 |
|  |  | **mutant** | 0.59 | 0.37 | 0.55 | 0.01 | 0.81 | 0.77 | 0.94 | 0.00 | 0.06 | 0.72 | 0.00 | 1.00 |
|  |  | **mixed** | 0.05 | 0.17 | 0.06 | 0.00 | 0.00 | 0.04 | 0.01 | 0.00 | 0.08 | 0.15 | 0.06 | 0.00 |

**n:** total number of alleles
