# Supplementary material for: Genetic diversity and signatures of selection of drug resistance in Plasmodium populations from both human and mosquito hosts in continental Equatorial Guinea
Source: Malar J. 2013 Mar 27;12:114. doi: 10.1186/1475-2875-12-114 (PMC3621214; doi:10.1186/1475-2875-12-114)
Supplement: Additional file 4 — Pfdhfr point mutations and their respective STR haplotypes in allele size. [file 1475-2875-12-114-S4.docx]

**Additional file 4.** *Pfdhfr* point mutations and their respective STR haplotypes in allele size.

| **Villages** | **Haplotype** | **Point mutation** | **Allele size (bp)** | | | **N** |
| --- | --- | --- | --- | --- | --- | --- |
|  |  |  | ***locus* 0.8kb** | ***locus* 4.3kb** | ***locus* 7.7kb** |  |
|  | **K1** | **R59/N108** | 113 | 183 | 210 |  |
| **Ngonamanga** | **H1** | **N108** | 113 | 183 | 210 | 1 |
|  | **H2** | **I51/R59/N108** | 113 | 179 | 210 | 1 |
|  | **H3** | **I51/R59/N108** | 113 | 183 | 210 | 32 |
|  | **H4** | **I51/R59/N108** | 113 | 183 | 214 | 1 |
| **Miyobo** | **H1** | **N108** | 113 | 183 | 210 | 2 |
|  | **H3** | **I51/R59/N108** | 113 | 183 | 210 | 6 |
|  | **H5** | **R59** | 113 | 183 | 210 | 1 |
|  | **H6** | **N108** | 107 | 183 | 200 | 1 |
|  | **H7** | **I51/N108** | 117 | 183 | 210 | 1 |
|  | **H8** | **I51/N108** | 113 | 183 | 210 | 1 |
|  | **H9** | **R59/N108** | 113 | 183 | 210 | 10 |
