# Supplementary material for: Genetic diversity and signatures of selection of drug resistance in Plasmodium populations from both human and mosquito hosts in continental Equatorial Guinea
Source: Malar J. 2013 Mar 27;12:114. doi: 10.1186/1475-2875-12-114 (PMC3621214; doi:10.1186/1475-2875-12-114)
Supplement: Additional file 5 — Pfdhps point mutations and their respective STR haplotypes in allele size. [file 1475-2875-12-114-S5.docx]

**Additional file 5.** *Pfdhps* point mutations and their respective STR haplotypes in allele size.

| **Village** | **Haplotype** | **Point mutation** | **Allele size (bp)** | | | **N** |
| --- | --- | --- | --- | --- | --- | --- |
|  |  |  | ***locus* 0.8kb** | ***locus* 4.3kb** | ***locus* 7.7kb** |  |
|  | **K1** | **A437G** | 131 | 103 | 108 |  |
| **Ngonamanga** | **H1** | **A436/G581** | 117 | 105 | 124 | 1 |
|  | **H2** | **A436/G581** | 117 | 109 | 118 | 1 |
|  | **H3** | **G437/G581** | 113 | 107 | 110 | 1 |
|  | **H4** | **G437/G581** | 115 | 103 | 120 | 1 |
|  | **H5** | **G437/G581** | 121 | 109 | 112 | 1 |
|  | **H6** | **G437/G581** | 123 | 107 | 110 | 1 |
|  | **H7** | **G437/G581** | 131 | 117 | 126 | 2 |
|  | **H8** | **A436/G437/G581** | 121 | 109 | 112 | 4 |
|  | **H9** | **A436/G437/G581** | 133 | 103 | 120 | 1 |
| **Miyobo** | **H10** | **G581** | 117 | 109 | 124 | 1 |
|  | **H11** | **G581** | 117 | 105 | 126 | 2 |
|  | **H12** | **G581** | 117 | 117 | 126 | 1 |
|  | **H13** | **G581** | 121 | 111 | 118 | 1 |
|  | **H14** | **A436/G581** | 117 | 109 | 126 | 1 |
|  | **H15** | **A436/G581** | 123 | 103 | 126 | 1 |
|  | **H16** | **G437/G581** | 117 | 103 | 138 | 1 |
|  | **H17** | **G437/G581** | 117 | 105 | 120 | 2 |
|  | **H18** | **G437/G581** | 117 | 105 | 138 | 1 |
|  | **H19** | **G437/G581** | 121 | 107 | 110 | 1 |
|  | **H20** | **G437/G581** | 121 | 107 | 114 | 2 |
|  | **H5** | **G437/G581** | 121 | 109 | 112 | 1 |
|  | **H21** | **G437/G581** | 123 | 109 | 112 | 1 |
|  | **H22** | **G437/G581** | 123 | 107 | 110 | 3 |
|  | **H23** | **G437/G581** | 125 | 107 | 114 | 1 |
|  | **H24** | **G437/G581** | 135 | 103 | 120 | 1 |
|  | **H25** | **A436/G437/G581** | 121 | 107 | 114 | 1 |
